# A novel genomic classification system of gastric cancer via integrating multidimensional genomic characteristics

**DOI:** 10.1007/s10120-021-01201-9

**Published:** 2021-06-06

**Authors:** Haiyong Wang, Yongfeng Ding, Yanyan Chen, Junjie Jiang, Yiran Chen, Jun Lu, Mei Kong, Fan Mo, Yingying Huang, Wenyi Zhao, Ping Fang, Xiangliu Chen, Xiaodong Teng, Nong Xu, Yimin Lu, Xiongfei Yu, Zhongqi Li, Jing Zhang, Haohao Wang, Xuanwen Bao, Donghui Zhou, Ying Chi, Tianhua Zhou, Zhan Zhou, Shuqing Chen, Lisong Teng

**Affiliations:** 1 Department of Surgical Oncology, The First Affiliated Hospital, Zhejiang University School of Medicine, Hangzhou, 310003 China; 2 Department of Medical Oncology, The First Affiliated Hospital, Zhejiang University School of Medicine, Hangzhou, 310003 China; 3 Department of Pathology, The First Affiliated Hospital, Zhejiang University School of Medicine, Hangzhou, 310003 China; 4 Innovation Institute for Artificial Intelligence in Medicine and Zhejiang Provincial Key Laboratory of Anti-Cancer Drug Research, College of Pharmaceutical Sciences & Alibaba-Zhejiang University Joint Research Center of Future Digital Healthcare, Zhejiang University, Hangzhou, 310058 China; 5 Hangzhou Neoantigen Therapeutics Co., Ltd., Hangzhou, 310051 China; 6 Alibaba-Zhejiang University Joint Research Center of Future Digital Healthcare, Alibaba DAMO Academy, Hangzhou, 311121 China; 7 Institute of Gastroenterology, Zhejiang University, Hangzhou, 310058 China; 8 Institute of Drug Metabolism & Pharmaceutical Analysis & Zhejiang Provincial Key Laboratory of Anti-Cancer Drug Research, College of Pharmaceutical Sciences, Zhejiang University, Hangzhou, 310058 China

**Keywords:** Gastric cancer, Molecular classification, Whole-exome sequencing, Genomic landscape, Precision oncology

## Abstract

**Background:**

Gastric cancer (GC) is one of the leading causes of cancer deaths with high heterogeneity. There is currently a paucity of clinically applicable molecular classification system to guide precise medicine.

**Methods:**

A total of 70 Chinese patients with GC were included in this study and whole-exome sequencing was performed. Unsupervised clustering was undertaken to identify genomic subgroups, based on mutational signature, copy number variation, neoantigen, clonality, and essential genomic alterations. Subgroups were characterized by clinicopathological factors, molecular features, and prognosis.

**Results:**

We identified 32 significantly mutated genes (SMGs), including *TP53, ARID1A, PIK3CA, CDH1,* and *RHOA*. Of these, *PREX2, PIEZO1,* and *FSIP2* have not been previously reported in GC. Using a novel genome-based classification method that integrated multidimensional genomic features, we categorized GC into four subtypes with distinct clinical phenotypes and prognosis. Subtype 1, which was predominantly Lauren intestinal type, harbored recurrent *TP53* mutation and *ERBB2* amplification, high tumor mutation burden (TMB)/tumor neoantigen burden (TNB), and intratumoral heterogeneity, with a liver metastasis tendency. Subtype 2 tended to occur at an elder age, accompanying with frequent *TP53* and *SYNE1* mutations, high TMB/TNB, and was associated with poor prognosis. Subtype 3 and subtype 4 included patients with mainly diffuse/mixed type tumors, high frequency of peritoneal metastasis, and genomical stability, whereas subtype 4 was associated with a favorable prognosis.

**Conclusions:**

By integrating multidimensional genomic characteristics, we proposed a novel genomic classification system of GC associated with clinical phenotypes and provided a new insight to facilitate genome-guided risk stratification and disease management.

**Supplementary Information:**

The online version contains supplementary material available at 10.1007/s10120-021-01201-9.

## Introduction

Gastric cancer (GC) is a common malignancy with high rates of morbidity and mortality worldwide, and nearly two-thirds of GC cases and deaths occur in Asia [1]. At present, China has the largest number of GC patients, in which more than 679,000 new GC patients are diagnosed and about 498,000 GC-related deaths occur annually [2, 3]. GC patients are frequently diagnosed at advanced stages with a low 5-year survival rate. Peritoneal dissemination and liver metastasis are the most important causes of treatment failure [4]. In addition to surgery, chemotherapy, and targeted therapies, immunotherapy has recently been considered as one of the promising treatments for specific subtypes of GC [4, 5]. However, high heterogeneity of GC remains a crucial barrier to optimal treatment strategies for a better survival benefit [4, 6].

Next-generation sequencing (NGS) has dramatically expanded the knowledge of the molecular basis of tumor heterogeneity. Similar to other types of cancer, multiple molecular classification systems of GC have been presented [7–9]. Among these studies, the most acknowledged one was The Cancer Genome Atlas (TCGA) classification. By integrating sequencing data from six molecular platforms, TCGA classified GC into four molecular subtypes, including Epstein–Barr virus (EBV), microsatellite instability (MSI), chromosomal instability (CIN), and genomically stable (GS). Notably, genomics was considered as a fundamental basis for simplifying multi-omics data in the TCGA’s study [7]. Other studies also demonstrated the critical role of genomics in precise molecular classification for various types of cancer [10, 11]. Different aspects of in-depth genome-based analyses, such as mutational signature (reflecting the characteristic imprints of mutagenic processes of human cancer) [12] and clonality (i.e., intratumor heterogeneity) [13], have been reported to be clinically relevant in multiple types of cancer. Neoantigens are mutated peptides derived from somatic mutations and represent excellent targets for immunotherapy, due to their specific expression in cancer tissue [14]. Recent advances in genomics and bioinformatics have facilitated identification of neoantigens through cancer genome sequencing [15, 16]. Our previous study preliminarily suggested the correlation between neoantigen load and clinicopathological variables of GC [17]. Therefore, it is promising to integrate these aspects and develop a novel genome-based classification of GC with an improved clinical significance. In addition, patients of TCGA cohort were predominately from Western population, but the epidemiological, histological, and molecular features of GC differ between Asian and Western countries [18–20]. Asian GC have higher prevalence of intestinal type tumors, lower prevalence of MSI GC, and more favorable prognosis. Therefore, an Asian-specific genomic classification is necessary.

In the present study, we performed whole-exome sequencing (WES) of 70 GC samples from Chinese population. With an innovative integration of genomic features, such as mutational signature, copy number variation (CNV), neoantigen, clonality, and essential genomic alterations, we divided GC samples into four subtypes according to distinct genomic features, clinicopathological characteristics, patterns of metastasis, and overall survival (OS). Besides, we proposed a new clinically relevant genome-based classification system for GC and provided rationale for genome-guided therapy.

## Methods

### Patient tissue samples

Primary GC patients who underwent gastrectomy at the First Affiliated Hospital of Zhejiang University School of Medicine between January 2016 and January 2018 were retrospectively procured. Cases were enrolled in this study according to the criteria as follows: at least 18 years old; pathologically confirmed gastric adenocarcinoma; complete clinicopathological and follow-up data; without autoimmune disease or other cancer types; without previous chemotherapy/radiotherapy. The pathologic diagnoses and characteristics were independently determined by at least two experienced pathologists (Mei Kong and Xiaodong Teng). The tumor-node-metastasis (TNM) Staging was according to AJCC 8th edition [21]. The follow-up data were obtained by medical record system, phone, and letter every 3 to 6 months from the date of surgery. A written informed consent was obtained prior to participation for each participant. This study was approved by the ethics committee of the First Affiliated Hospital of Zhejiang University School of Medicine.

### DNA extraction and whole-exome sequencing

DNA was extracted from 70 GC tumors and paired normal gastric mucosa. QIAamp DNA Mini Kit (Qiagen) was used to isolate genomic DNA from tumor tissues and matched normal mucosa according to the manufacturer’s instructions. Then we combined the following two steps to verify the quality of isolated genomic DNA. First, DNA degradation and contamination were monitored on 1% agarose gels. Second, Qubit® DNA Assay Kit in Qubit® 2.0 Flurometer (Invitrogen, USA) was used to quantify DNA concentration.

Whole-exome library construction was generated using the Agilent SureSelect Human All Exon V6 Kit (Agilent Technologies, Santa Clara, CA, USA). The index-coded samples were clustered on a cBot Cluster Generation System using Hiseq PE Cluster Kit (Illumina). Then the DNA libraries were sequenced on Illumina Hiseq platform (Illumina, San Diego, California, USA) and 150 bp paired-end reads were generated. We first conducted data quality control and then performed all downstream bioinformatics analyses based on the high-quality clean data, in which reads containing an adapter, reads containing poly-N, and low-quality reads were removed. The paired-end clean reads were aligned to the Human Genome Reference Consortium build 37 (GRCh37) using BWA v.0.7.8 [22]. Mapped reads were then de-duplicated using Sambamba tools (v0.4.7) [23].

### Somatic mutation detection and significantly mutated genes identification

Identification of somatic single-nucleotide variants (SNVs) was conducted by muTect (v 1.1.4) [24], and the somatic InDels were detected by Strelka (v1.0.13) [25]. ANNOVAR (ANNOVAR_2015Mar22) [26] was used to annotate variant call format files. The Mutational Significance in Cancer (MuSic, version: Genome-Model-Tools-Music-0.04) algorithm [27] was used to identify significantly mutated genes (SMGs) from the profiles of somatic SNVs and InDels (False discovery rate (FDR) < 0.25).

### Mutation signature analysis

Mutational signatures were characterized according to the 96-substitution classification. Based on the frequency of 96 mutation types, Nonnegative Matrix Factorization (NMF) method (v0.22) was performed to extract mutational signatures and compare them with 30 known signatures referenced in the Catalogue of Somatic Mutations in Cancer (COSMIC) database using SomaticSignatures packages (v2.24.0) [28]. The similarity of mutation signatures was evaluated with cosnine similarity > 0.9, which suggested common signatures. Signatures 1, 6, 17, and 29 were identified in our samples. Then unsupervised hierarchical clustering was performed to identify the clusters of mutational signatures (Sig-cluster) according to the proportional contribution of each signature per sample.

### Copy number analysis

Somatic copy number variations (SCNVs) were identified using CNVkit [29]. Then GISTIC 2.0 (v 2.0.22) [30] was used to identify the genome regions with significant alterations and screen out the recurrent CNV regions (parameters: -rx 0 -ext xls -fname ALL -ta 0.1 -td 0.1 -js 4 -qvt 0.25 -cap 1.5 -board 1-maxseg 2000 -conf 0.99 -genegistic 1 -armpeel 1 -brlen 0.7 -gcm extreme -savegene 1). In addition, unsupervised hierarchical clustering was performed to identify the clusters of copy-number variations (CNV-cluster) based on discretized CNVs.

### HLA genotyping and neoantigen prediction

The raw data of WES were processed by software TSNAD (available on http://github.com/jiujiezz/TSNAD) [31]. This software developed by our research group combines multiple algorithms to identify somatic mutations, determine HLA genotyping, and predict neoantigens. After identification of somatic mutations, TSNAD can determine HLA genotyping by SOAP-HLA [32]. Subsequently, NetMHCpan [33] was invoked to predict mutation-derived neoantigens which could bind to class I MHC molecules. Then unsupervised hierarchical clustering was conducted to identify the neoantigen clusters (NEA-cluster) according to the neoantigen number. Since we lack expression data, there might be over-prediction of neoantigens to some extent.

### Analysis of clonal architecture of somatic mutations

The R package SciClone [34] (http://github.com/genome/sciclone) was used to infer the clonal and subclonal architecture of somatic mutations by analyzing the variant allele frequencies in an individual sample using the Bayesian binomial mixture model. SciClone parameters: minimumDepth = 20, maximumClusters = 10, copyNumberMargins = 0.25. Variant allele frequencies were clustered and visualized as previously reported [34]. The clusters of clonality (Clonality-cluster) was defined according to the clonal patterns.

### Integrative clustering

The consensus clustering was used to uncover molecular subtypes according to multidimensional genomic features, including Sig-cluster, CNV-cluster, NEA-cluster, Clonality-cluster, essential SMGs, and CNVs. Essential SMGs were selected with the overlap between the genes with a mutation frequency ≥ 10% in our cohort and the cancer-related genes in COSMIC [35] (https://cancer.sanger.ac.uk/cosmic/). Essential CNVs were selected with the overlap among the genes with alteration frequency ≥ 10%, the cancer-related genes in COSMIC and the genes involved in the key pathways in our cohort. The values of these variables are shown in the Table S1. Clustering was then done in R software (version 3.6.1) based on Euclidean distance using Ward's method.

### Integrated pathway analysis

Geneset of cancer-driving genes from Cancer Gene Census (CGC) was accessed from the COSMIC. Subsequently, according to the genetic alteration rates of the cancer-driving genes in our cohort, we performed KEGG pathway enrichment analysis using DAVID bioinformatics Resource 6.8.

### Immunohistochemical staining

In order to evaluate PD-L1 protein expression in gastric cancers, formalin-fixed, paraffin-embedded (FFPE) tissue sections were retrieved from tumor block of our GC cohort. 4um paraffin sections were routinely deparaffinized and rehydrated. Antigen retrieval was performed and endogenous peroxidase, non-specific protein binding sites were blocked and subsequently incubated in primary antibodies overnight at 4 ℃. PD-L1 immunohistochemical (IHC) was performed using rabbit anti-human PD-L1 (clone MXR003, MXB biotechnologies, Fujian China), which recognizes an epitope in the PD-L1 cytoplasmic domain and reactivity confirms the full-length PD-L1 protein expression. PD-L1 staining was observed in tumor cells and adjacent immune cells. Combined Positive Score (CPS) methods was introduced in our IHC study. Tumor cells, adjacent lymphocytes and phagocytes with positive membrane staining were counted as positive cells, and CPS ≥ 5 was annotated as positive staining (representatives were shown in Fig. S1) [36]. Observation and scoring was performed by an expert immunopathologist (Mei Kong).

### Statistical analysis

The statistical analysis was performed by SPSS 21.0 software. The differences between categorical variables were compared by Pearson’s Chi-square test or Fisher’s exact test. The continuous or non-parametric variables were assessed by one-way ANOVA or Mann–Whitney test, respectively. Survival data were analyzed by Kaplan–Meier curves with log-rank test. Univariate and multivariate Cox regression analysis were performed to calculate the hazard ratio and 95% confidence interval. A *P* value of < 0.05 was considered significant.

## Results

### Patients’ clinicopathological features

Paired fresh-frozen tumor tissue and adjacent normal tissue were collected from a cohort of 70 pathologically confirmed GC patients (ZJU-GC) (Table S2). Among the patients, 47 (67.1%) were males and their median age at the time of diagnosis was 60 years (range 38–80 years old). The majority (61.4%) of the patients were diagnosed with TNM stage III or stage IV. Besides, 51.4% of cases were identified as intestinal-type and 57.1% were with poor differentiation. During the follow-up, 21.4% of cases developed peritoneal dissemination and 15.7% of cases developed liver metastasis.

### Landscape of somatic mutations of GC

**Fig. 1:**
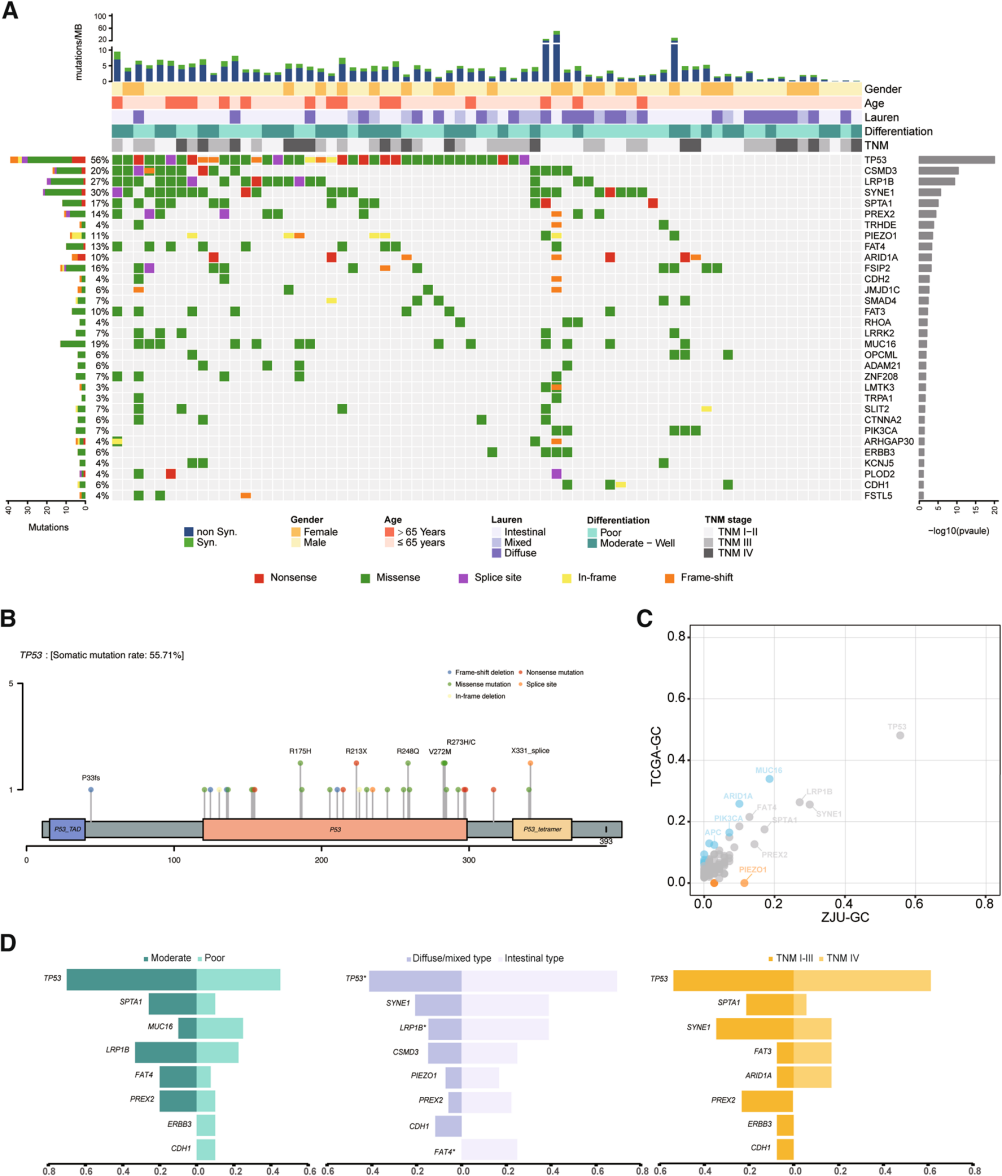
The landscape of somatic mutations in ZJU-GC cohort. **A** Somatic mutations of 70 paired samples in ZJU-GC cohort. The middle matrix shows the somatic mutations by gene (row) and by sample (column). The top histogram shows the number of non-synonymous and synonymous mutations in each individual sample. The top tracks show the clinicopathological characteristics, including gender, age, Lauren types, differentiation, and TNM stage. The left histogram shows the number of somatic mutations accumulated on 70 ZJU-GC samples in each gene. The right histogram shows p values of each gene calculated from MuSic analysis. **B** Distribution of non-synonymous TP53 somatic mutations identified. **C** Comparison of gene mutation rate between ZJU-GC cohort and TCGA-GC. Orange or blue dots represent the genes with significantly higher or lower mutation rate, respectively. **D** Comparison of gene mutation rates by clinicopathological subtypes

WES was conducted on 70 matched normal-tumor pairs from GC patients with a mean depth of 314- and 176-fold, respectively (Table S3). A total of 57,580 somatic mutations were identified from all samples (10,506 were of non-synonymous and 3560 were of synonymous) (Tables S4 and S5). In addition, 32 genes were identified as significantly mutated genes (SMGs) by the MuSiC algorithm (Fig. 1A). Of these, 14 were reported as SMGs in at least one previous GC genome sequencing study, such as *TP53, ARID1A, PIK3CA, CDH1, RHOA,* and *SMAD4* (Table S6). However, 18 mutations have not been previously reported in GC samples (e.g., *PREX2, PIEZO1,* and *FSIP2*). Distribution of nonsynonymous somatic *TP53* mutations is illustrated in Fig. 1B, and 87.2% occurred in a DNA-binding domain. The distribution of mutation sites for other three recurrent SMGs (*CSMD3, LRP1B,* and *SYNE1*) is also presented in Fig. S2. The mutational frequencies of the majority of SMGs were similar to those in TCGA-GC, whereas *ARID1A* and *PIK3CA* were mutated at remarkably lower frequencies in ZJU-GC (23% vs. 10% and 18% vs. 8%, Fig. 1C, Table S7), and the frequency of *PIEZO1* mutation in ZJU-GC cohort was significantly higher than that in TCGA-GC (12% vs. 0%, *P* < 0.01). Notably, *LRP1B* was the more frequent mutant in intestinal type tumors (*P* = 0.03, Fig. 1D, Table S8) and tended to be less frequent in those with poor differentiation (*P* = 0.052). In addition, mutations of *FAT4* were exclusive in intestinal type tumors (25% vs. 0%, *P* = 0.002).

### KEGG pathway analysis and targetable genes

**Fig. 2:**
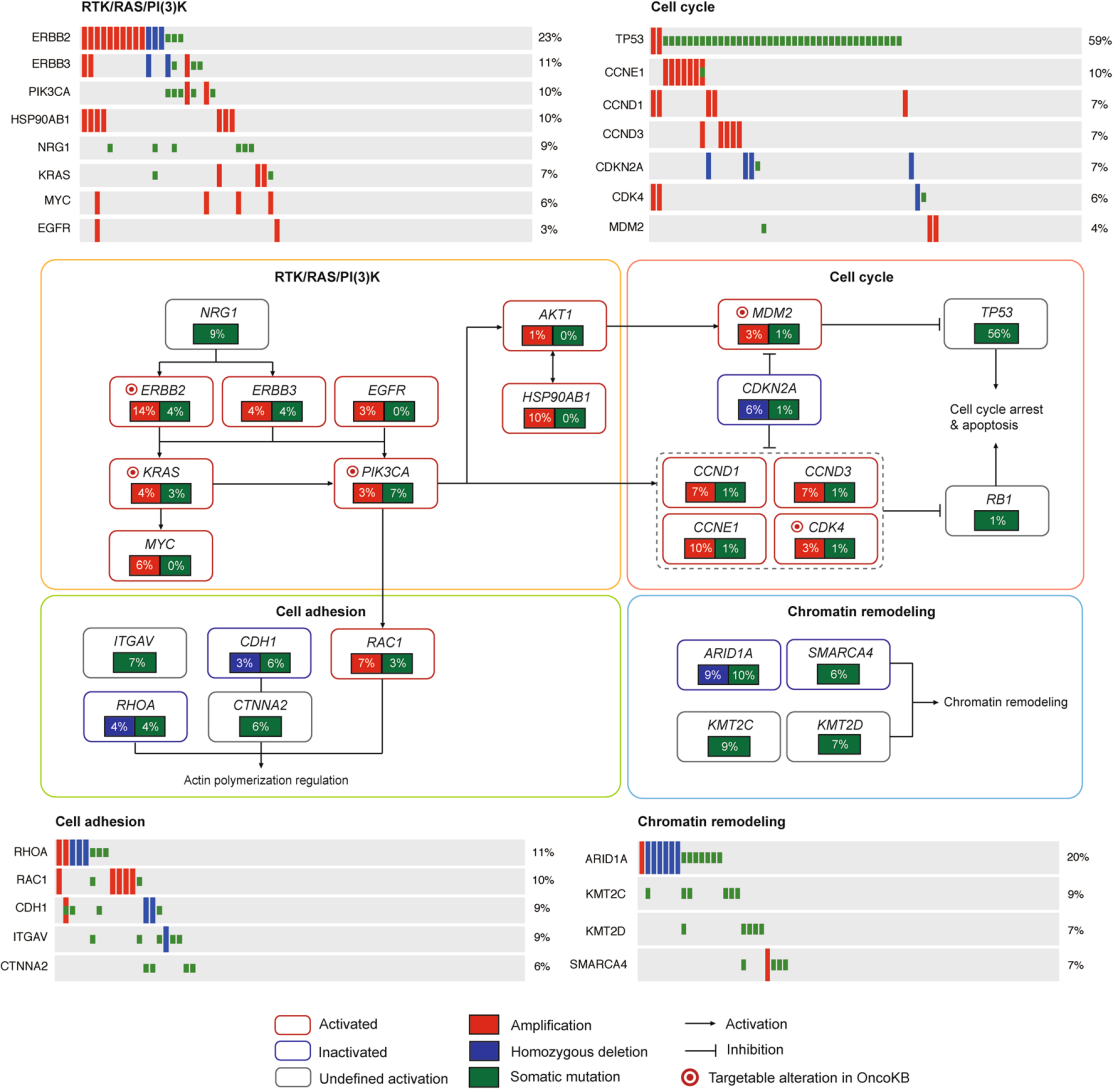
Genomic alterations of signaling pathways in ZJU-GC cohort. Gene somatic mutations and copy number variations are characterized in key signaling pathways, including RTK/RAS/PI(3)K pathway, cell adhesion pathway, cell cycle pathway, and chromatin remodeling pathway. Genes are grouped by the pathways and linked by the line and arrows showing molecular interactions

Kyoto Encyclopedia of Genes and Genomes (KEGG) pathway analysis of somatic mutations and somatic CNV revealed altered signaling pathways in GC, including RTK/RAS/PI(3)K, p53/cell cycle, cell adhesion, and chromatin remodeling (Fig. 2). The RTK/RAS/PI(3)K pathway was primarily altered by amplifications of *ERBB2* (14%), *HSP90AB1* (10%), and *MYC* (6%), as well as *PIK3CA* mutation (7%). P53/cell cycle pathway was frequently altered mainly owing to recurrent *TP53* mutations (56%) and amplifications of cyclin-encoding genes (*CCNE1* (10%), *CCND1* (8%), and *CCND3* (8%)). Other frequently altered genes included those involved in focal adhesion (*RHOA, ITGAV*), adherens junction (*CDH1*) or chromatin remodeling (*ARID1A, SMARCA4, KMT2C, and KMT2D*). In addition to the well-established target, ERBB2 amplification, other alterations, such as *PIK3CA* mutation and *CDK4* amplification, could serve as potential therapeutic targets. According to the OncoKB database (https://www.oncokb.org), 31 out of 70 patients harbored at least one potentially targetable gene alteration (Table S9).

### Identification and characterization of multidimensional genomic features

#### Mutational signatures

**Fig. 3:**
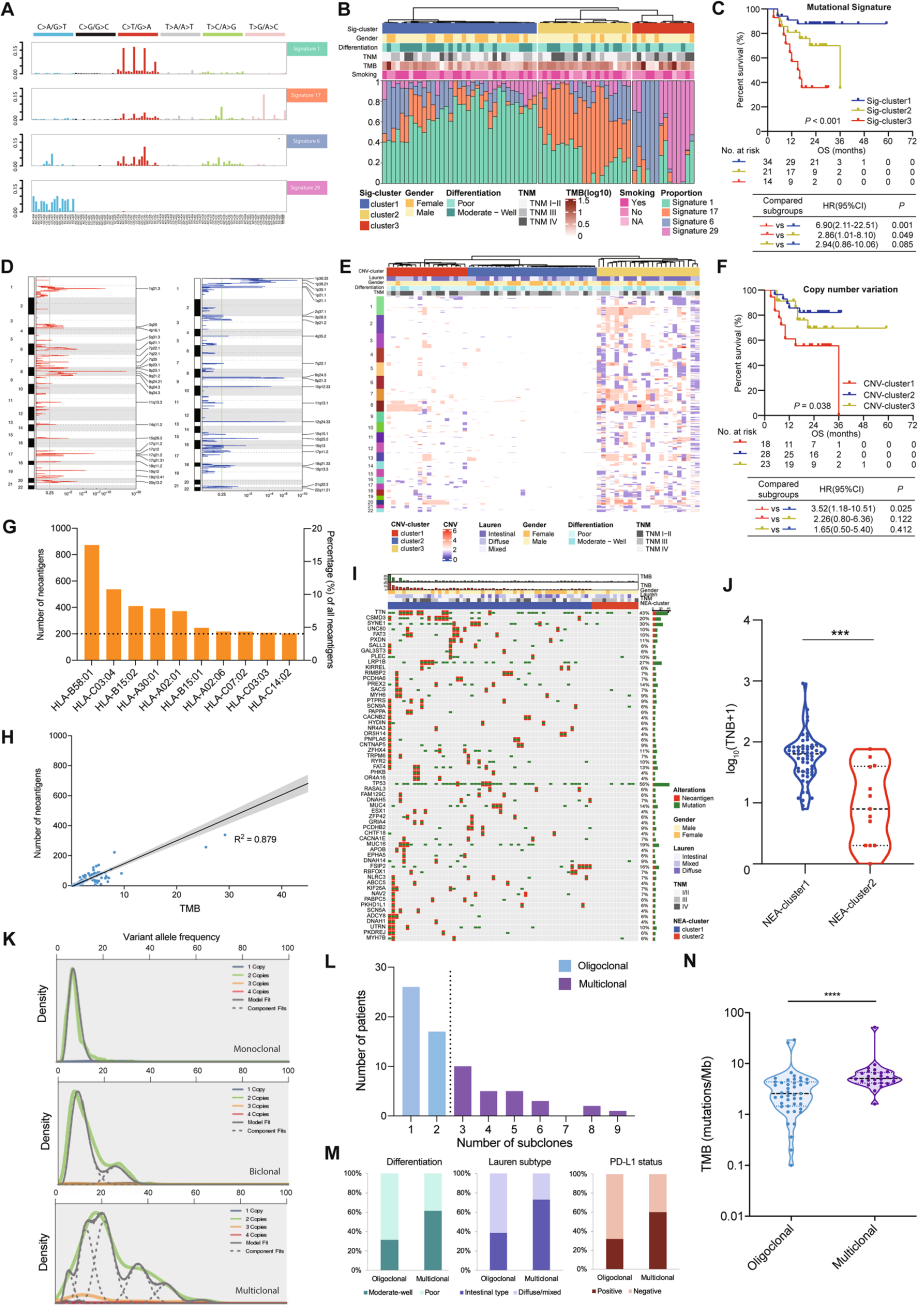
Characterization and identification of genomic features including mutational signatures (**A**–**C**), copy number variations (**D**–**F**), predicted neoantigen (**G**–**J**), and clonality (**K**–**N**) in ZJU-GC cohort. **A** Mutational signatures are characterized according to the 96-substitution classification, with horizontal axis showing mutation types of 96 substitutions and vertical axis showing the estimated mutations of each mutation type. **B** Unsupervised clustering of mutational signatures for 70 GC samples. **C** Association between three clusters of mutational signatures and OS in ZJU-GC cohort. **D** GISTIC 2.0 significant CNVs with amplifications on left and deletions on right. **E** Unsupervised clustering of CNVs for 70 GC samples. **F** Association between three clusters of CNVs and OS. **G** Predicted combining site of neoantigen. **H** Correlation between TMB and TNB in ZJU-GC cohort. **I** Unsupervised clustering of predicted neoantigens and somatic mutations in 70 ZJU-GC samples. The middle matrix shows the predicted neoantigens and somatic mutations by gene (row) and by sample (column). The top histograms show TNB and TMB. The right histogram shows the number of predicted neoantigens and somatic mutations accumulated on 70 GC samples in each gene. According to the status of predicted neoantigens, 70 samples are divided into two clusters. **J** Comparison of TNB between NEA-cluster 1 and NEA-cluster 2. **K** Identification of tumor clonality. **L** Classification of tumor clonality in ZJU-GC. According to the clonal status, 70 samples are divided into two clonality groups: oligoclonal group and multiclonal group. **M** Comparison of histological characteristics between oligoclonal group and multiclonal group, including differentiations, Lauren type, and PD-L1 status. **N** Comparison of TMB between oligoclonal group and multiclonal group. Sig-cluster: mutational signature cluster; CNV-cluster: copy number variation cluster; NEA-cluster: neoantigen cluster; TMB: tumor mutation burden; TNB: tumor neoantigen burden; OS: overall survival. ^***^P < 0.001, ^****^ *P* <0.0001

The predominant somatic mutation types were C:G > T:A transitions and C:G > A:T transversions (Fig. S3). A total of four independent mutational signatures, COSMIC Signature 1 (initiated by spontaneous deamination of 5-methylcytosine associating with aging [12]), Signature 6 (associated with deficient DNA mismatch repair [12, 37]), Signature 17 (attributed to oxidative stress [38] and associated with a poor prognosis [38]), and Signature 29 (as a tobacco-related signature [39]) could be identified (Fig. 3A, Table S10). Using unsupervised clustering, GC samples were divided into three subtypes (Sig-cluster1, Sig-cluster2, and Sig-cluster3), according to the proportional contribution of each signature per sample (Fig. 3B). We further investigated the association between these subtypes and OS. The Sig-cluster1 subtype had the longest OS, while Sig-cluster3 had the shortest (log-rank *P* < 0.001, Fig. 3C). Subsequently, multivariate Cox regression analyses also indicated a significant difference in prognosis among those subtypes (Fig. S4, Table S11).

#### Somatic CNVs

Forty-two significant focal CNVs (24 with amplification and 18 with deletion) were identified using GISTIC 2.0 (Fig. 3D, Table S12). The recurrent CNVs with amplification included *CCNE1* (19q12), *ERBB2* (17q12), *CCND1* (11q13.3), *MUC4* (3q29), *VEGFA* (6q21.1), and *NOTCH1* (9q34.3). Significantly deleted CNVs included 1p35.3, 1p21.1, 3p21.2, 9p21.3, 16q21, 17q12, and 19p13.3, encompassing a great number of tumor suppressor genes, including *ARID1A, MLH1, CDKN2A, CDH1, BRCA1, TP53,* and *SMARCA4*. Based on significantly altered CNVs per sample, 70 GC patients were clustered into three subtypes by unsupervised clustering (CNV-cluster1, CNV-cluster2, and CNV-cluster3; Fig. 3E). We further observed that the three subtypes were significantly interrelated to prognosis (*P* = 0.038, Fig. 3F) and associated with the patients’ age at diagnosis, gender, and the Lauren subtype (Table S13).

#### Predicted neoantigens derived from mutated genes

A total of 4994 predicted neoantigens were identified in 70 patients (Table S14). The number of predicted neoantigens ranged from 0 to 918 (median, 52). Besides, there were 873 (17.5%) neoantigen affinities to allele HLA-B58:01 (Fig. 3G, Table S15). The number of neoantigens was significantly correlated with tumor mutational burden (TMB) (*R*^2^ = 0.879, *P* < 0.001, Fig. 3H). Neoantigens were frequently derived from somatic mutations of *TTN* (13%), followed by *CSMD3* (10%) (Table S16). Unsupervised clustering according to predicted neoantigens categorized GC into two clusters (Fig. 3I) with significantly different tumor neoantigen burden (TNB) (*P* < 0.001, Fig. 3J). The associations between these two clusters and clinicopathological characteristics are presented in Table S17 and Fig. S5.

#### Clonal and subclonal architectures

SciClone was applied to specimens to reconstruct the clonal and subclonal architectures, reflecting the intratumor heterogeneity. Patterns of clonality included monoclonal (a single dominant clone with or without one minor subclone), biclonal (two major clones), and complex clonal patterns (more than two clones (Fig. 3K)). We defined GC with monoclonal and biclonal patterns as *oligoclonal* and those with complex clonal pattern as *multiclonal* (Fig. 3L). Comparison of clinicopathological features of these two subtypes showed that multiclonality was significantly associated with well or moderate differentiation, the Lauren intestinal type, and programmed death-ligand 1 (PD-L1) positivity (Fig. 3M and Table S18). GC cases with multiclonal features carried significantly higher TMB (Fig. 3N).

### Integrative genomic classification of GC

**Fig. 4:**
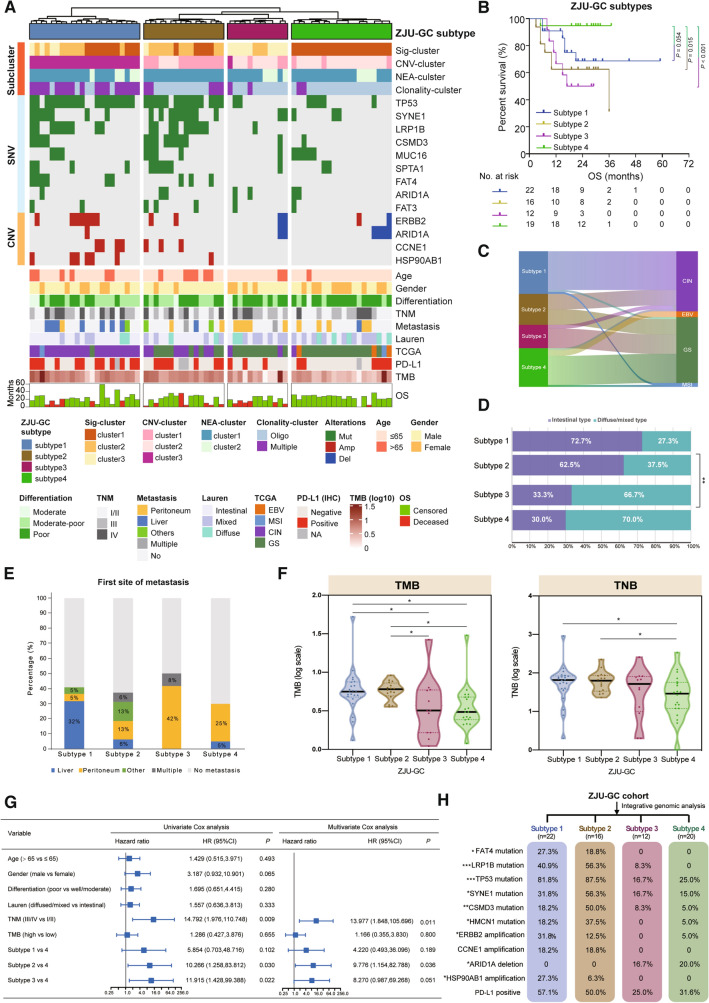
Integrated genomic classification of gastric cancer in association with clinicopathological features and patient outcomes. **A** Unsupervised clustering of integrated genomic features (Sig-cluster, CNV-cluster, NEA-cluster, clonality-cluster), frequent mutated genes, and copy number variations. 70 GC samples are divided into four ZJU-GC subtypes: subtype 1 (blue), subtype 2 (brown), subtype 3 (rose red), and subtype 4 (green). Clinicopathological and molecular characteristics are depicted at the bottom. **B** Association between ZJU-GC subtypes and OS. **C** Sankey diagram showing the association between ZJU-GC subtypes and TCGA subtypes. **D** Association between ZJU-GC subtypes and Lauren types. **E** Comparison of first-metastasis site among ZJU-GC subtypes. **F** Comparison of TMB and TNB among ZJU-GC subtypes. **G** Forest plot showing univariate and multivariate Cox regression analysis for the association between ZJU-GC subtype and OS. **H** Molecular characteristics of four ZJU-GC subtypes. Sig-cluster: mutational signature cluster; CNV-cluster: copy number variation cluster; NEA-cluster: neoantigen cluster; TMB: tumor mutation burden; TNB: tumor neoantigen burden; OS: overall survival; CIN: chromosomal instability; EBV: Epstein-Barr virus; MSI: microsatellite instability; GS: genomically stable. ^*^ *P* < 0.05

To identify genomic classification of GC associated with patients’ clinicopathological features and survival outcomes, unsupervised clustering was conducted based on subgroups derived from mutational signatures, CNVs, predicted neoantigens, clonality, as well as essential gene alterations. As shown in Fig. 4A, 70 GC cases were divided into four subtypes (ZJU-GC subtypes): subtype 1 (*n* = 22), subtype 2 (*n* = 16), subtype 3 (*n* = 12), and subtype 4 (*n* = 20). Subsequently, we investigated the association of the four subtypes with OS (Fig. 4B). We found that subtype 4 had the longest OS, while subtype 2 and subtype 3 both had significantly the shortest OS than subtype 4, and subtype 1 had a moderate OS. Thereafter, associations between the four subtypes and TCGA molecular subtypes or Lauren histological types were further explored. We observed that 90.9% of tumors in subtype 1 were classified into CIN subtype, whereas subtype 2 contained 50.0% CIN type and 50.0% GS type (Fig. 4C, Table S19). The majority of tumors in subtype 3 (83.3%) and subtype 4 (75.0%) were classified into GS type. Notably, GC tumors in subtype 1 and subtype 2 had a significantly higher proportion of intestinal histology compared with subtype 3 and subtype 4 (*P* < 0.01, Fig. 4D). Furthermore, when examining the first site of metastasis, we found that subtype 1 harbored the highest rate of liver metastasis in comparison with other subtypes (*P* < 0.01), whereas subtype 3 and subtype 4 developed significantly higher rate of peritoneal metastasis in comparison with other subtypes (*P* < 0.05) (Fig. 4E, Fig. S6). In addition, those in subtype 1 and subtype 2 harbored higher TMB compared with those in the other two subtypes, as well as higher TNB than that in subtype 4 (Fig. 4F). To further explore the prognostic value of the proposed classification system, multivariate Cox regression analysis was undertaken, and it was found that the classification system was an independent prognostic factor after adjusting for TNM stage and TMB (Fig. 4G). Four subtypes displayed distinct molecular features (Fig. 4H, Table S20). Subtype 1 harbored recurrent mutations in TP53 (81.8%), as well as amplification of *ERBB2* (31.8%) and *HSP90AB1* (27.3%). Subtype 2 was enriched in frequent mutations of *TP53* (87.5%), *LRP1B* (56.3%), and *SYNE1* (56.3%). Subtype 3 and subtype 4 both exhibited frequent deletion of *ARID1A* (subtype 3, 16.7%; subtype 4, 20.0%).

**Fig. 5:**
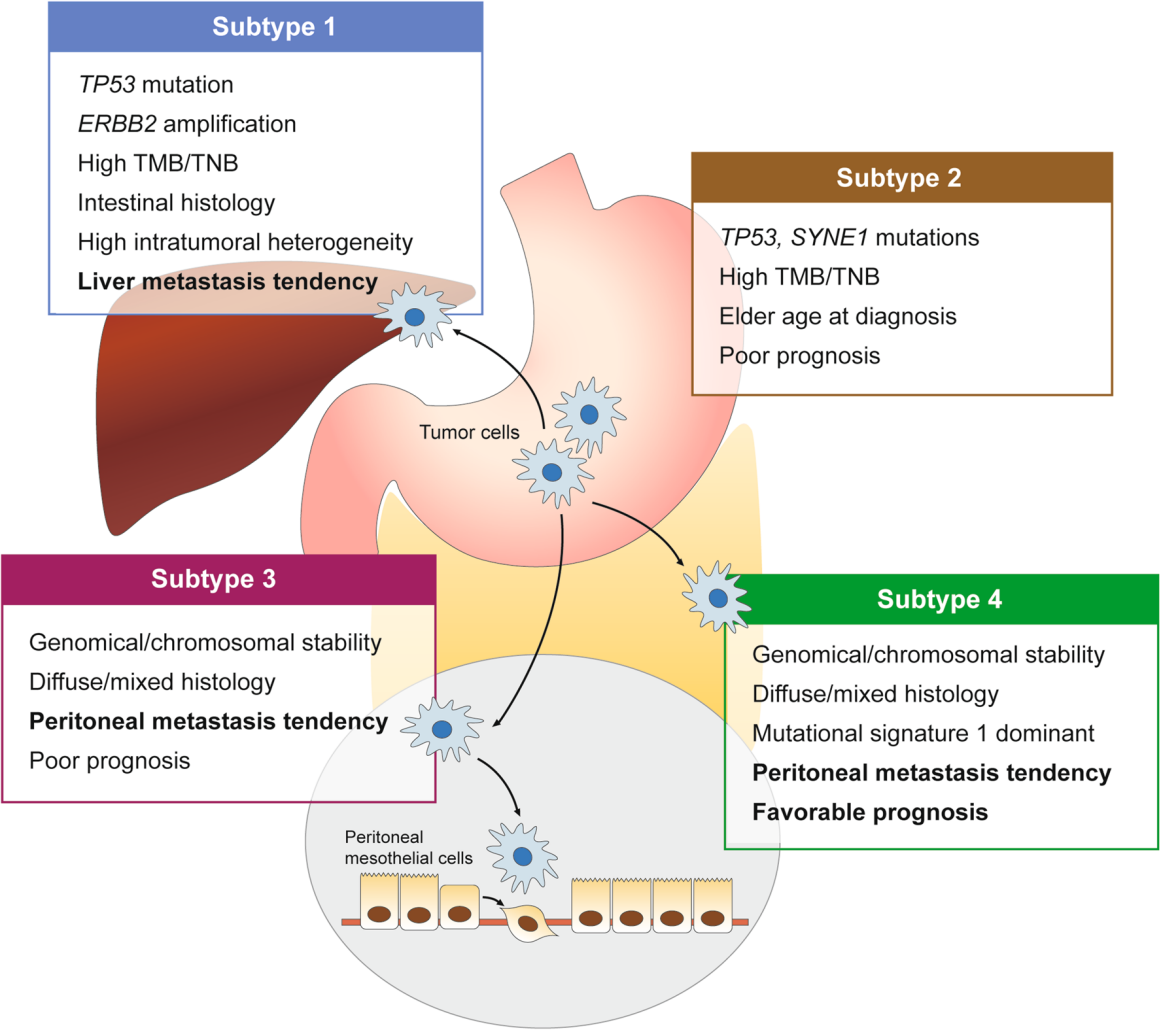
Summary of key features of gastric cancer in four genomic subtypes. The schematic shows the salient characteristics associated with each of four ZJU-GC subtypes

In summary, we proposed a new genomic classification system, including four subtypes associated with distinct metastatic patterns and prognosis (Fig. 5). Subtype 1, which is predominantly Lauren intestinal type, harbored recurrent *TP53* mutation and *ERBB2* amplification, TMB/ TNB, intratumoral heterogeneity, and has a liver metastasis tendency. Subtype 2 tends to occur at an elder age, accompanying with high *TP53* and *SYNE1* mutations, high TMB/TNB, and is associated with poor prognosis. Subtype 3 and subtype 4 include GC patients with mainly diffuse/mixed type tumors, high frequency of peritoneal metastasis, and genomical/chromosomal stability, whereas subtype 4 is associated with a favorable prognosis.

### Independent cohort validation of the genomic classification

In order to increase the clinical applicability of our genomic classification system, we performed dimensionality reduction analysis, and five features (Sig-cluster, CNV-cluster, FAT4 mutation, LRP1B mutation, and CCNE1 CNV) were selected. Then we constructed a simplified genomic classification model to predict subtypes based on naive Bayes algorithm (details were shown in supplementary methods and Fig. S7). Tenfold cross-validation of this prediction model was performed and internal validation accuracy was up to 95.7%.

WES was performed in 23 GC tumors and paired normal gastric mucosa from an independent cohort (supplementary methods and Table S21–22). After genomic classification prediction using the model based on five selected genomic features, we achieved predicted ZJU-GC subtype for each sample in the validation cohort (Table S22). 23 GC cases were divided into four subtypes: subtype 1 (*n* = 10), subtype 2 (*n* = 5), subtype 3 (*n* = 3), and subtype 4 (*n* = 5). Similar to ZJU-GC cohort, the genomic subtype showed significant association with metastasis pattern in the validation cohort that subtype 1 harbored the highest rate of liver metastasis while subtype 3 and subtype 4 showed higher rate of peritoneal metastasis (Fig. S8A-B). Further analysis revealed that GC cases of subtype 1 had higher prevalence of Lauren intestinal histology and those of subtype 3 and subtype 4 were enriched in diffuse/mixed type (Fig. S8C). In addition, subtype 1 and subtype 2 harbored higher TMB and *SYNE1* mutation compared with the other two subtypes. Subtype 1 was enriched in *TP53* mutation and *ERBB2* amplification (Fig. S8D–E). Therefore, our validation suggested that the molecular classification had good expansibility and robustness and confirmed the relevance between genomic subtypes and clinicopathological features.

## Discussion

In the current study, we characterized the landscape of genomic alterations in Chinese GC patients. Based on mutational signature, CNV, predicted neoantigen, clonality analysis, and essential genomic alterations, we presented four subtypes of GC associated with different clinical phenotypes. The current study added further layers of information about the genomic characteristics and molecular classification of GC and could lead to improvement in patient stratification, prognostication, and genome-guided therapy.

Based on the results of WES, we identified the SMGs in the current cohort. Among 32 SMGs identified, 14 (e.g., *TP53, SPTA1*) were reported as SMGs in at least one of 9 previous GC-based studies [40–48], while 18 genes, such as *PREX2, PIEZO1, TRHDE, FSIP2,* and *FAT3*, have not been previously reported. Among them, the most frequently mutated gene was *TP53* in the current study, which encoded the tumor suppressor and transcription factor p53. Previous genomic-based studies confirmed the high frequency of *TP53* mutations in GC samples (40–50%) [8, 40], which is consistent with the present study (56%). To date, no drug abrogating the oncogenic functions of p53 mutant has been approved for the treatment of cancer [49]. A recent study suggested the potential of arsenic trioxide in treating p53-mutated cancer patients [50]. Importantly, in the current research, 2 ZJU-GC subtypes (subtypes 1 and 2) were enriched for *TP53* mutations (81.8% and 87.5%, respectively, Fig. S9), suggesting that they may benefit from *TP53*-targeted therapy. Spectrin Alpha, Erythrocytic 1 (*SPTA1*) encodes a member of a family of molecular scaffold proteins, linking the plasma membrane to the actin cytoskeleton. *SPTA1* mutation is associated with a variety of hereditary red blood cell disorders, such as hereditary elliptocytosis and hereditary spherocytosis [51]. In the present study, *SPTA1* was identified as a SMG for GC, which was consistent with two previous genomic-based studies [42, 45]. In addition, in a recent study on Mongolians with hepatocellular carcinoma, *SPTA1* was reported as a potential driver gene [52]. However, whether *SPTA1* is a driver gene in GC and how *SPTA1* mutation mechanistically contributes to tumorigenesis remained elusive. Phosphatidylinositol-3,4,5-Trisphosphate Dependent Rac Exchange factor 2 (*PREX2*) belongs to PREX family and is an important regulator of Purkinje cell morphogenesis and motor coordination [53]. Although *PREX2* was not reported as a SMG or a driver gene in previous GC-based studies, it was reported as a SMG in a study on melanoma [54], as well as being a candidate driver gene of pancreatic carcinogenesis [55]. A recent study on 46 patients with advanced GC who received anti-programmed cell death protein 1 (PD-1) antibody revealed that *PREX2* mutation was correlated with a poor progression-free survival [56]. Understanding the role of key mutations in GC tumorigenesis and progression are promising aspects in future research.

In the decision tree of TCGA classification derived from analysis of six molecular platforms, DNA-level features, such as EBV, MSI, and the degree of SCNVs could substantially differentiate into four distinct subgroups [7]. Li et al. identified two molecular subtypes of non-hypermutated GC that correlated with prognosis by SMG analysis [57]. To the best of our knowledge, we first proposed an integrated genome-based classification system, including mutational signature, clonality, and neoantigen features. Mutational signatures have been attributed to specific carcinogenic factors with clinical relevance in multiple types of cancer [12, 58–60]. Clonality, which reflects intratumor heterogeneity, is associated with cancer aggressiveness, treatment resistance, and patient prognosis [13, 44]. Tumor-specific neoantigen, derived from accumulation of somatic mutations, has been proposed as a predictive indicator for response to immune checkpoint inhibitors [61–64].

We proposed a novel genome-based classification system with significant clinical relevance and prognostic value. Subtype 4 was found to be associated with significantly longer OS than the other subtypes, whereas subtype 2 and subtype 3 showed a relatively poorer prognosis, and subtype 1 had a moderate prognosis. In the TCGA classification system, studies showed that EBV and MSI had a better prognosis [65, 66], while the difference in OS between GS and CIN subtypes was found inconsistent in different cohorts [8, 67]. Compared with the original prognosis trend of our classification system, classifying ZJU-GC cohort using the TCGA genomic scheme yielded a weaker association with the prognosis, where GS and CIN subtypes showed no significant difference in OS (Fig. S10), which is similar to the result of ACRG cohort [8]. Tumors in subtype 1 were mainly grouped into CIN type, while those in subtype 3 and subtype 4 were grouped into GS subtype. Notably, subtype 3 and subtype 4 showed a remarkable difference in OS in the present analysis; similar result was found between patients with peritoneal metastasis in the two subgroups (Fig. S11). In molecular level, the distinction of these two subtypes was mainly attributed to the distribution of mutational signatures, i.e., enrichment of signature 1 (known as clock-like mutational process) in subtype 4. Signature 1 is commonly found in various types of cancer and is associated with patients’ age at diagnosis [68], and it has been reported to be a negative prognostic factor in triple-negative breast cancer [69], while its clinical relevance in GC has not been reported yet. Multiple difference has also been found significant CNV between subtypes 3 and 4, and subtype 4 harbored significant amplifications in more regions than subtype 3 (Fig. S12, Table S23). The proportion of GS tumors in our cohort (49%) and another GC cohort in Asia (45%) [8] was higher than that in TCGA (24%). Differentiation of subtype 3 and subtype 4 could be clinically informative as it reflects heterogeneity within GS subtype in Chinese patients, though validation in larger cohort is required. Multiomic analysis might further suggest the underlying mechanism of the prognosis difference. The immunity features of the four subtypes were also described in the current study. Subtype 1 and subtype 2 harbored higher TMB/TNB, PD-L1 expression, as well as more frequently *LRP1B* and *SYNE1* mutations. These findings indicated that the two mentioned subtypes may have a better response to immune checkpoint inhibitors [62, 64, 70–72]. Nevertheless, further studies with larger sample size need to be carried out to confirm our findings.

Another key finding of this study is that we identified GC subtypes associated with patterns of metastasis in synchronous or metachronous metastatic GC, and this relevance was reproduced in the validation cohort. Peritoneal dissemination and liver metastasis are the major metastatic patterns of GC. To our knowledge, information on metastasis pattern has not been reported by TCGA-GC classification. In the present research, we examined the first site of metastasis regardless of surgical resection, and observed a significant tendency of liver metastasis in subtype 1, as well as a tendency of peritoneal metastasis in subtype 3 and subtype 4. Lee et al. reported that diffuse/mixed type was associated with a peritoneal recurrence, and the Lauren intestinal type was associated with hematogenous metastasis [73]. In the current study, in both ZJU-GC cohort and the validation cohort, subtype 1 was enriched in the Lauren intestinal type, and subtype 3 and subtype 4 were enriched in diffuse/mixed type. Among Koreans, the ACRG research also indicated the existence of subtypes with different metastatic tendency based on transcriptomic analysis [8]. The current study, for the first time, proposed a genomic classification that differentiates molecular subtypes associated with tendency to liver or peritoneal metastasis. On the molecular basis, subtype 1 had the highest prevalence of *HSP90AB1* and *ERBB2* amplifications among the four subtypes. HSP90AB1 was previously found to promote epithelial-mesenchymal transition via Wnt/β-catenin signaling pathway in GC progression [74]. A large-scale prospective study reported that ERBB2 positivity was significantly correlated to liver metastasis and absence of peritoneal metastasis [75]. Additionally, deletion of *ARID1A* was commonly found in subtype 3 and subtype 4, and it was revealed that reduced expression of the chromatin remodeling gene could promote migration and invasion of GC cells by downregulating E-cadherin expression [76]. Collectively, the proposed classification system can be informative to guide studies on molecular oncology. Nevertheless, the underlying mechanism of different site-specific patterns of metastasis among ZJU-GC subtypes requires further investigation through in vitro and in vivo experiments. Furthermore, our classification could also guide site-specific recurrence monitoring and treatments in clinical scenario. For instance, an evidence suggested that addition of pre-emptive intraperitoneal chemotherapy to gastrectomy reduces the risk of peritoneal recurrence for GC patients who are at high-risk of peritoneal metastasis [77, 78]. Moreover, an ongoing phase III trial is currently recruiting GC patients at high risk of peritoneal metastasis (T3/4, irrespective of nodal or peritoneal cytology status) [79]. The combination of clinicopathological factors and the proposed genomic classification system may lead to a better risk stratification of peritoneal metastasis.

There are some limitations in the current study that should be clearly declared. This is a single-center, retrospective study and the sample size is relatively limited. In addition, in spite of an obvious clinical significance of the newly proposed genomic classification, little is known about its underlying mechanism. Therefore, future prospective, multi-center, and large cohort studies are warranted to confirm our findings and to search for the molecular basis of the different clinical phenotypes (e.g., metastatic pattern). Although the proposed classification system is clinically significant, there are still obstacles towards broad clinical application due to the lack of large cohort validation. Further optimization of the classification algorithm and large cohort validation will be the next directions.

In summary, the present study revealed the genomic characteristics of Chinese GC patients and proposed a unique genomic classification system with significant clinical relevance. Through interpretation of genomic information, this study provided a rationale for research on GC and for molecular classification of multiple types of cancer to guide precision medicine.

## Supplementary Information

Below is the link to the electronic supplementary material.

Supplementary file1 (DOCX 2441 KB)

Supplementary file2 (XLSX 304 KB)

Supplementary file3 (DOCX 18 KB)
