# Supplementary material for: A novel genomic classification system of gastric cancer via integrating multidimensional genomic characteristics
Source: Gastric Cancer. 2021 Jun 6;24(6):1227–41. doi: 10.1007/s10120-021-01201-9 (PMC8502137; doi:10.1007/s10120-021-01201-9)
Supplement: Supplementary file 1 — Supplementary file1 (DOCX 2441 KB) [file 10120_2021_1201_MOESM1_ESM.docx]

**Supplementary Figure**

**
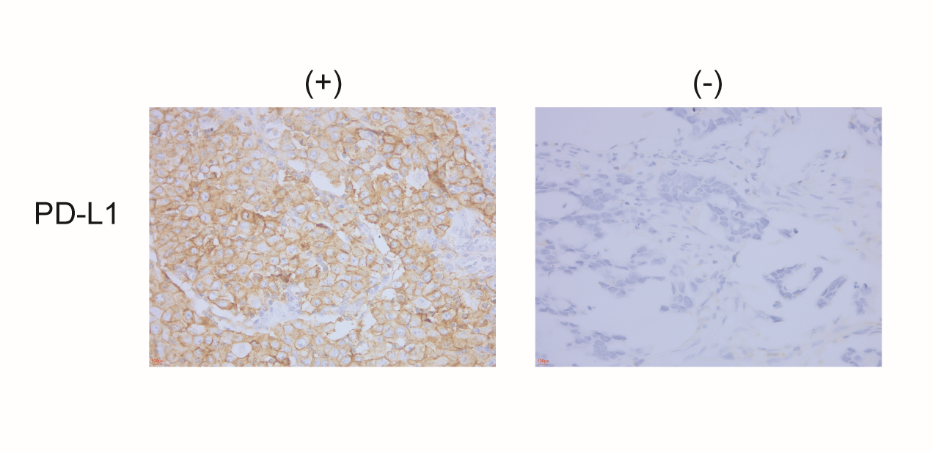
**

**Figure S1. Representatives of PD-L1 expression in immunohistochemical staining.** +, positive; -, negative.

**
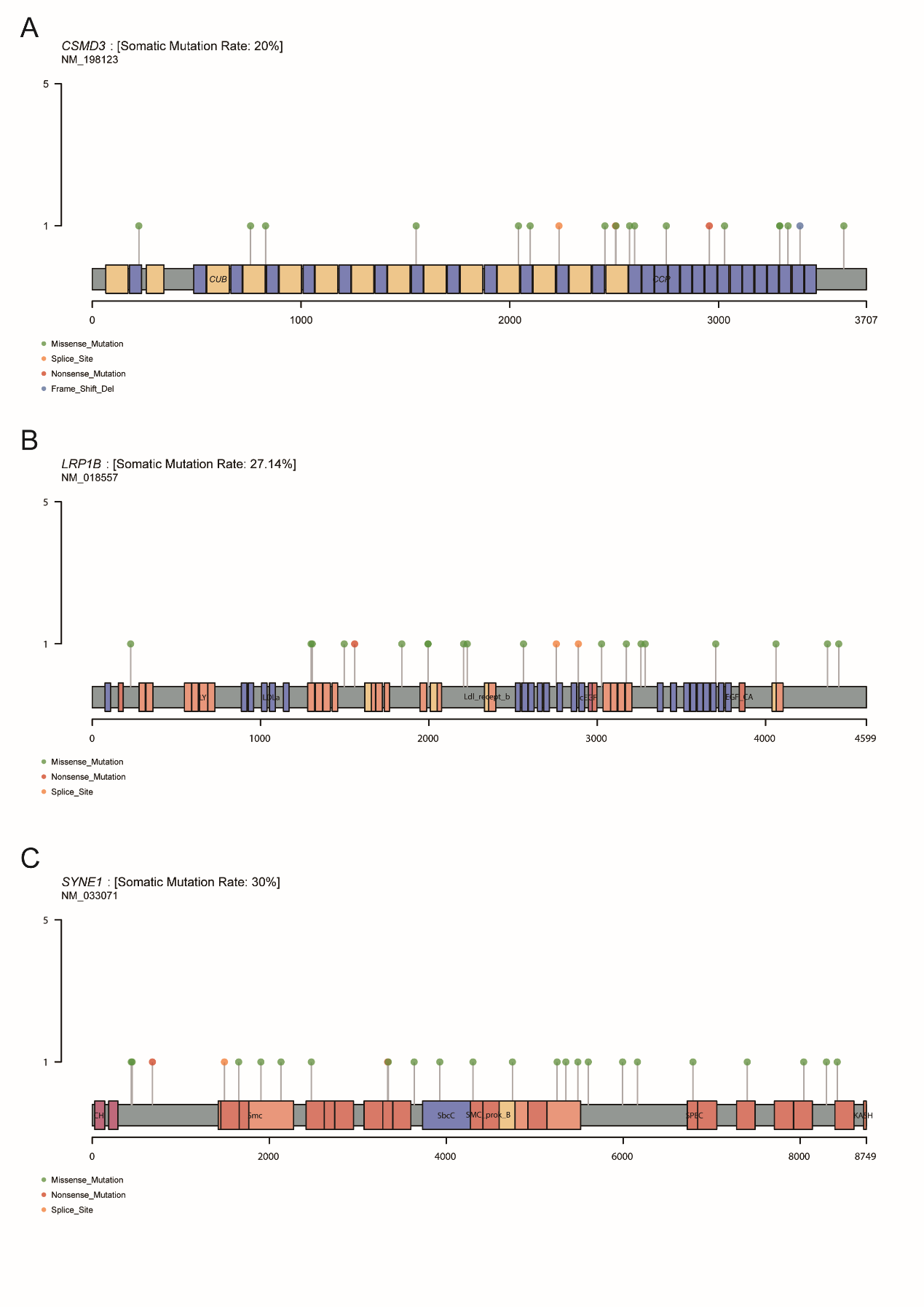
**

**Figure S2. Distribution of non-synonymous somatic mutations in frequently mutated genes in ZJU-GC.** **(A)** *CSMD3*; **(B)** *LRP1B*; **(C)** *SYNE1*.


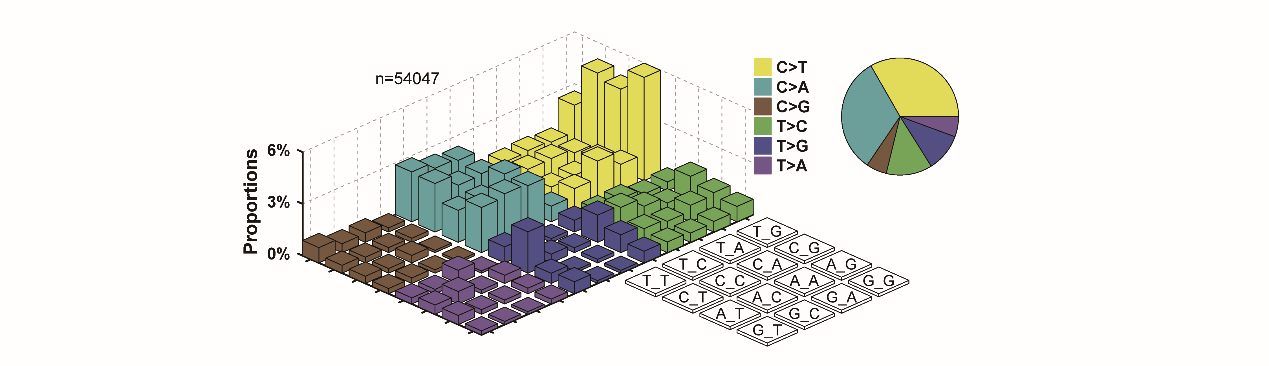


**Figure S3. Distribution of six base-substitution types in ZJU-GC.**


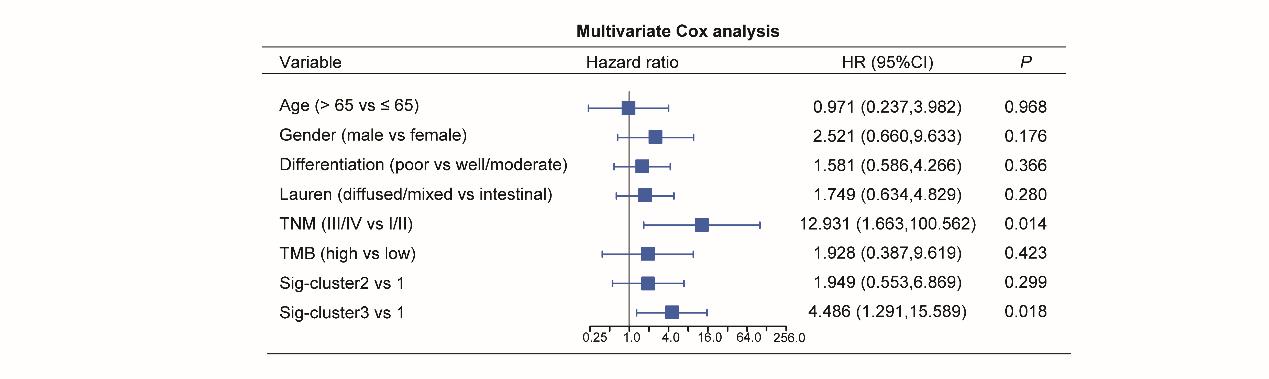


**Figure S4. Forest plot showing multivariate Cox regression analysis for the association between signature clusters and OS.** Sig-cluster, signature cluster; HR, hazard ratio; CI, confidence interval.

**
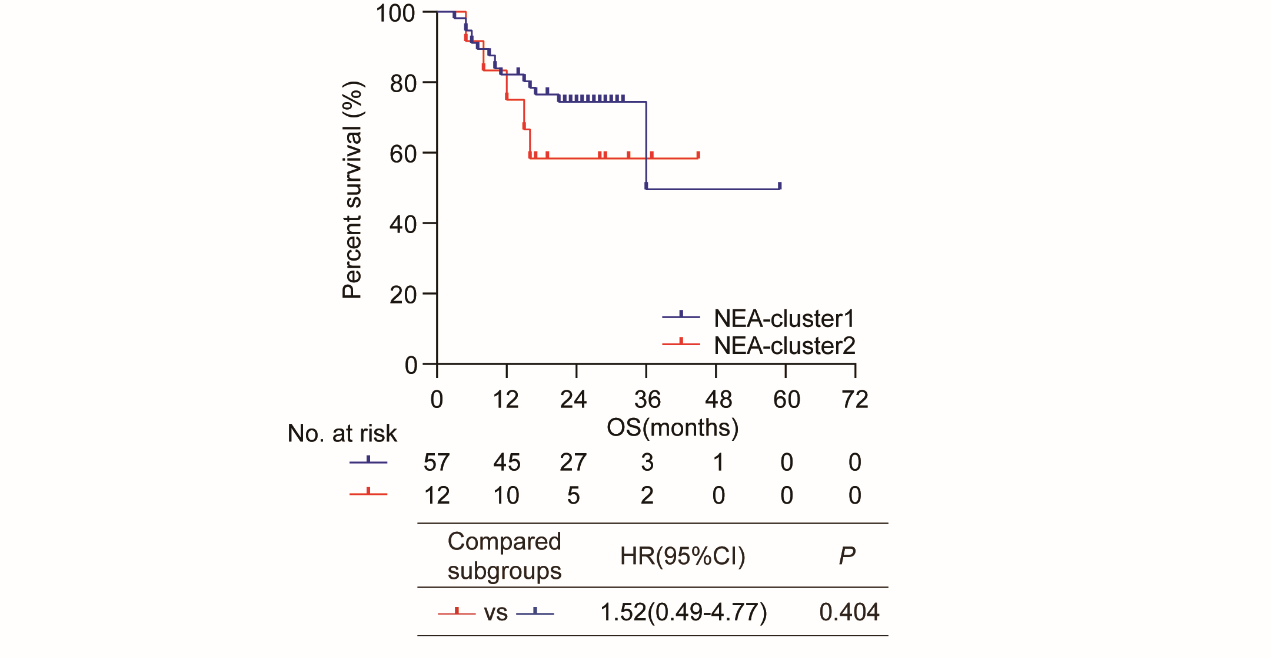
**

**Figure S5. Association between two clusters of neoantigens and OS in ZJU-GC cohort.** HR, hazard ratio; CI, confidence interval. Orange or blue dots represent the genes with significantly higher or lower mutation rate in ZJU-GC cohort, respectively.

**
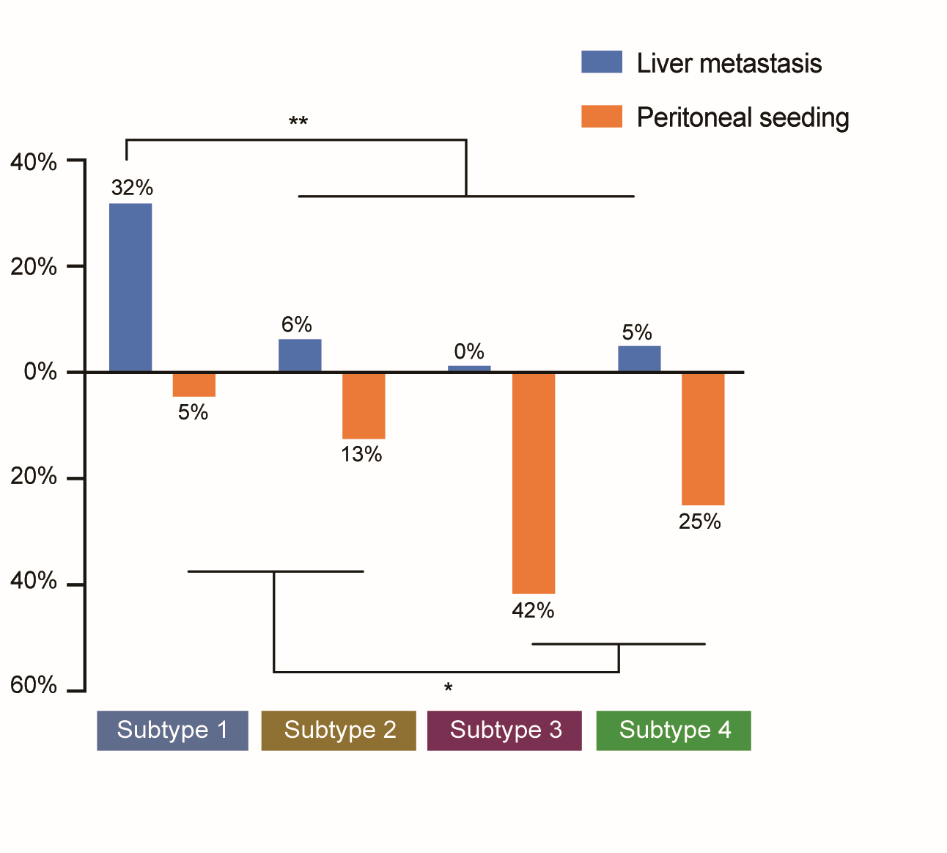
**

**Figure S6. Comparison of first-metastasis site among ZJU-GC subtypes.**

**
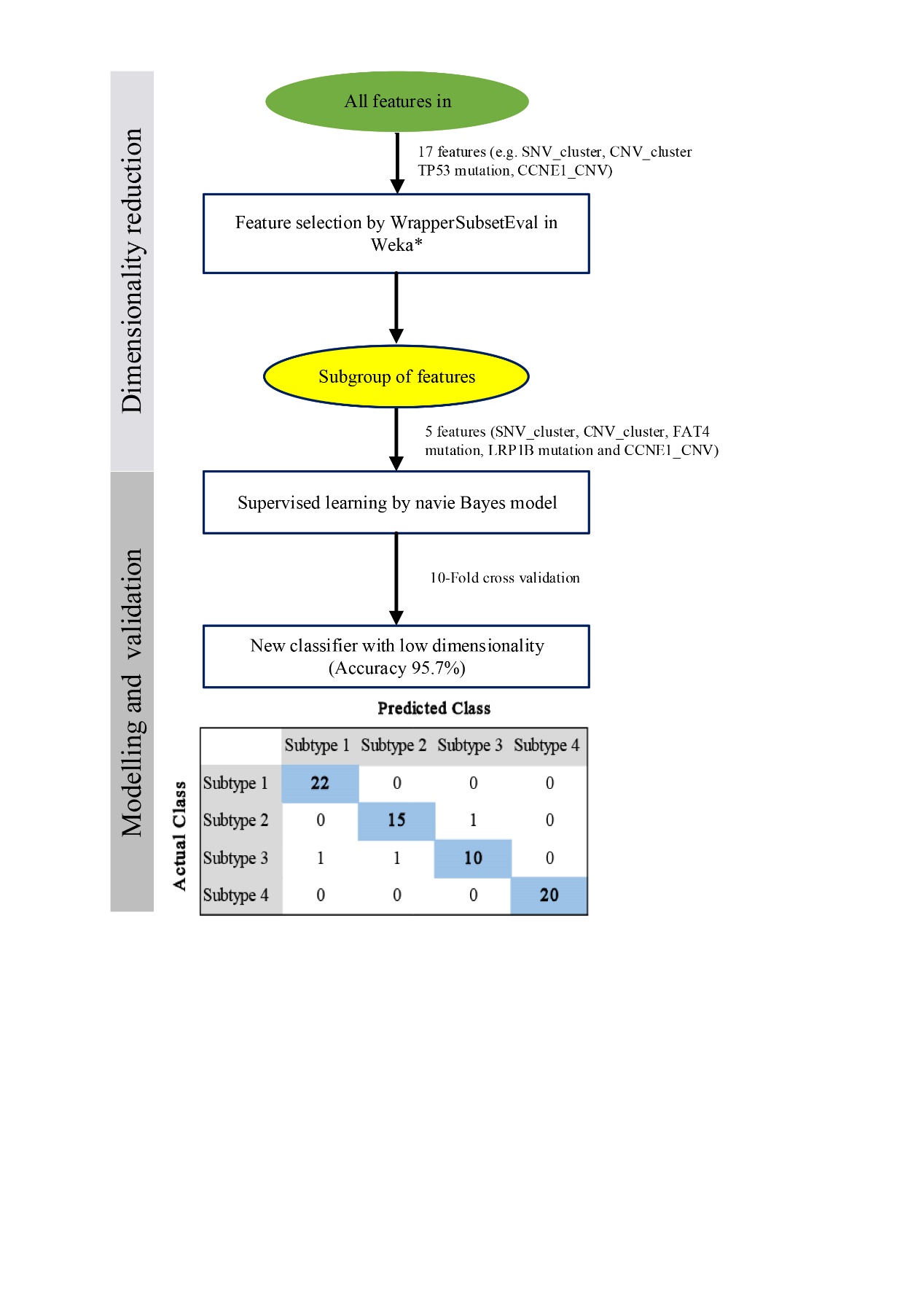
**

**Figure S7. Overview of workflow for dimensionality reduction, modelling and internal validation**. 17 features included Sig-cluster, CNV-cluster, NEA-cluster, Clonality-cluster, and 9 mutated genes (including TP53, SYNE1, LRP1B, CSMD3, MUC16, SPTA1, ARID1A, FAT3, and FAT4) and 4 CNVs (ERBB2, CCNE1, ARID1A, and HSP90AB1). *Weka is publicly available in https://www.cs.waikato.ac.nz/ml/weka/ as a complete industrial-strength software for machine learning.

**
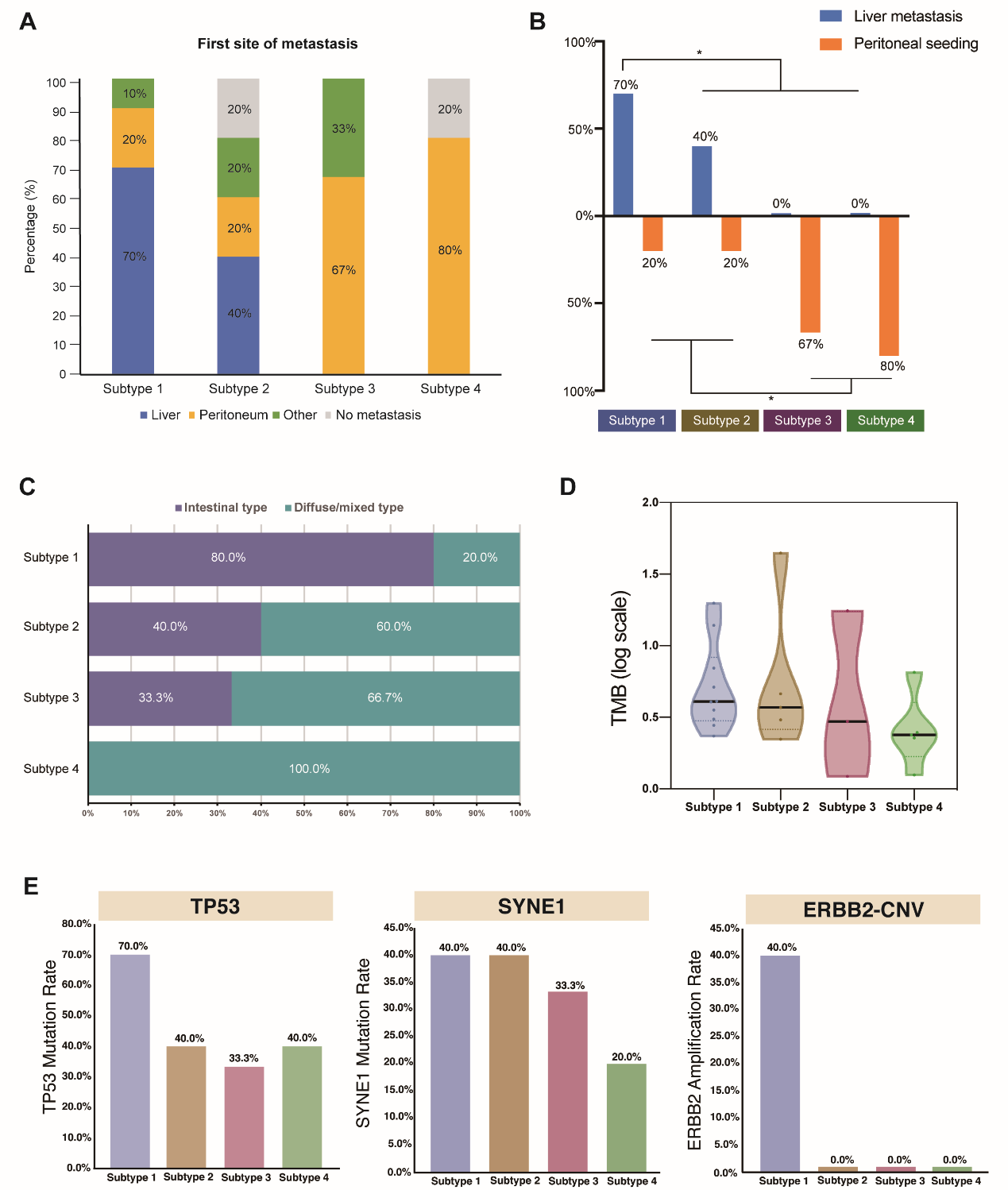
**

**Figure S8. Validation of the comparison of clinicopathological and genomic characteristics among ZJU-GC subtypes in an independent cohort. (A-B)** Comparison of first-metastasis site among ZJU-GC subtypes. **(C)** Association between ZJU-GC subtypes and Lauren types. **(D)** Comparison of TMB among ZJU-GC subtypes. **(E)** Comparison of key gene alterations among ZJU-GC subtypes, including TP53 mutation, SYNE1 mutation, and ERBB2 amplification.

**
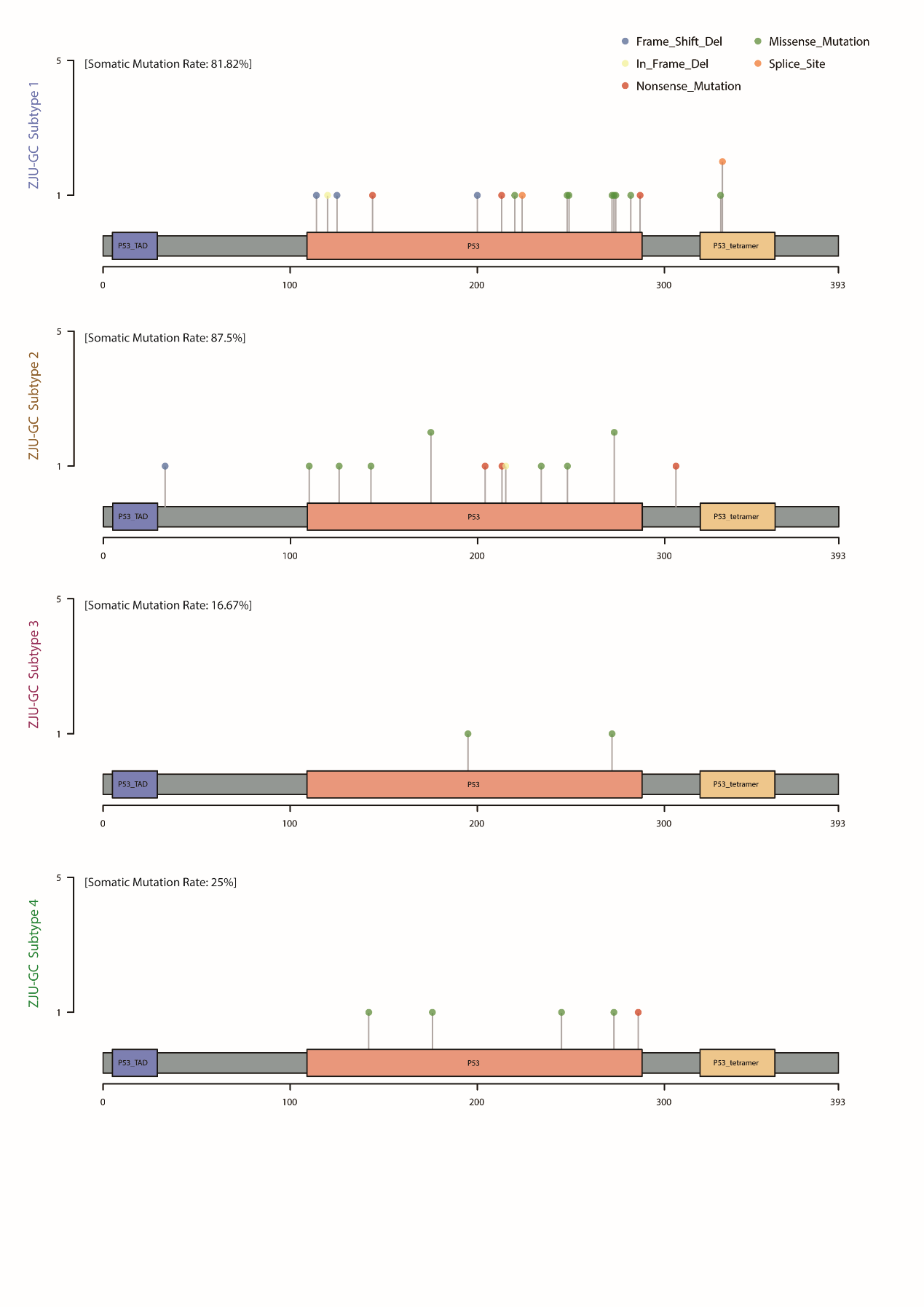
**

**Figure S9.** **Distribution of non-synonymous TP53 somatic mutations identified in four ZJU-GC subtypes.**

**
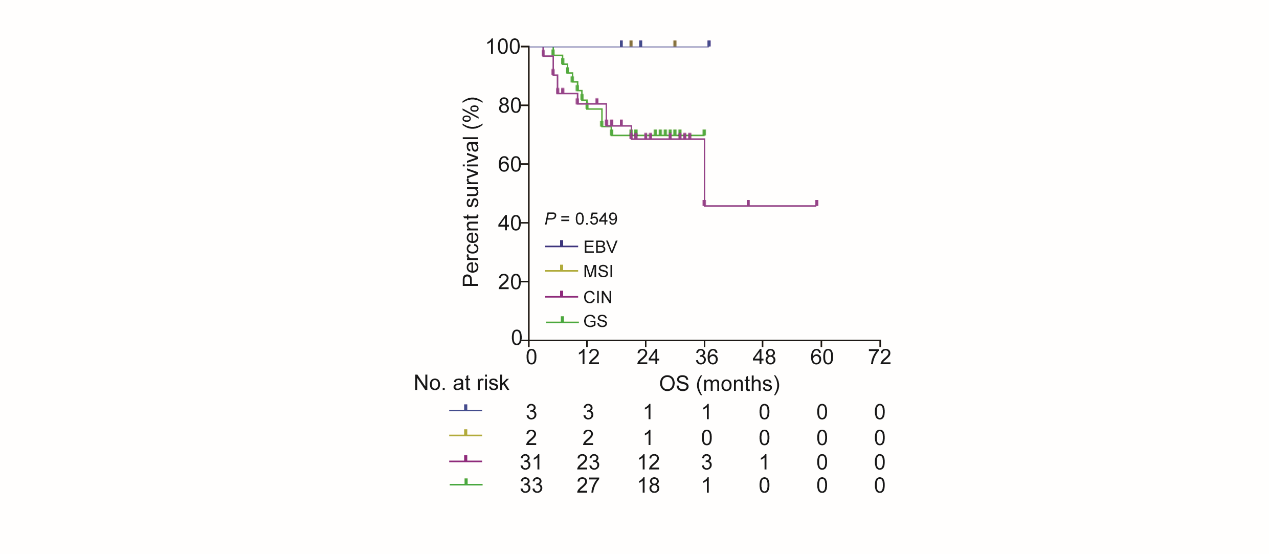
**

**Figure S10. Association between four TCGA subtypes and OS in ZJU-GC cohort.**

**
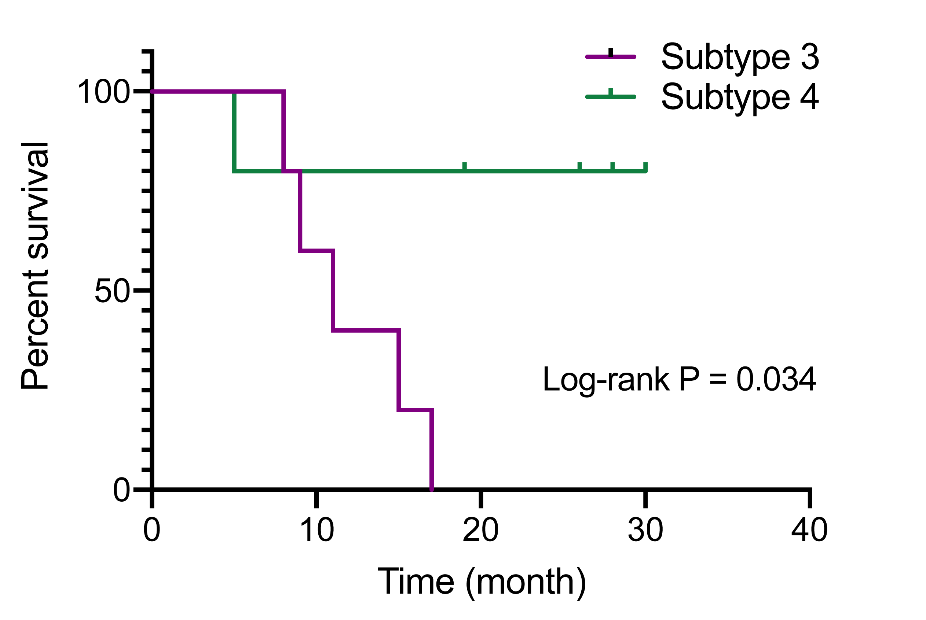
**

**Figure S11. Comparison of OS in patients with peritoneal metastasis between subtype 3 and subtype 4.**

**
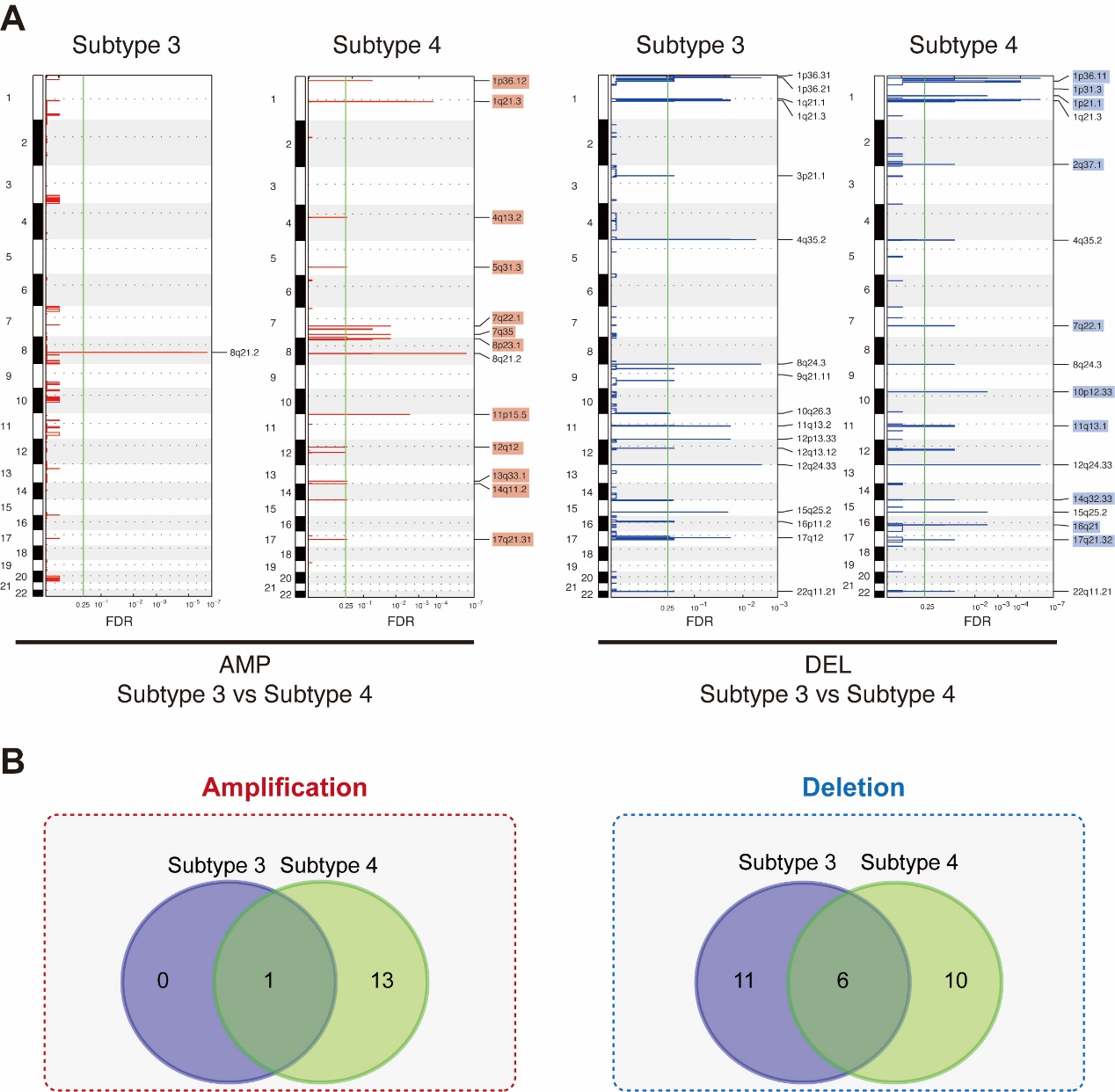
**

**Figure S12. Comparison of copy number alterations between subtype 3 and subtype 4. (A)** Distribution of copy number alterations in subtype 3 and subtype 4. **(B)** Venn analysis for copy number alterations in subtype 3 and subtype 4.
