# Supplementary material for: A novel genomic classification system of gastric cancer via integrating multidimensional genomic characteristics
Source: Gastric Cancer. 2021 Jun 6;24(6):1227–41. doi: 10.1007/s10120-021-01201-9 (PMC8502137; doi:10.1007/s10120-021-01201-9)
Supplement: Supplementary file 3 — Supplementary file3 (DOCX 18 KB) [file 10120_2021_1201_MOESM3_ESM.docx]

**Supplementary Methods**

**Dimensionality reduction, modelling and internal validation**

Firstly, all genomic features, including Sig-cluster, CNV-cluster, NEA-cluster, Clonality-cluster, essential SMGs and CNVs, used in consensus clustering were included and feature selection (dimensionality reduction) was performed by WrapperSubsetEval in Weka[1,2] (Figure S7). Weka is publicly available in <https://www.cs.waikato.ac.nz/ml/weka/> as a complete industrial-strength software for machine learning. Secondly, based on selected features, supervised learning by naive Bayes algorithm was performed and new classifier (concise genomic classification model with low dimensionality) was generated. In addition, the tenfold cross-validation method was used to calculate the accuracy of concise genomic classification model.

**Patient enrollment and follow-up of independent cohort**

Primary GC patients who underwent gastrectomy at the First Affiliated Hospital of Zhejiang University School of Medicine between January 2018 and October 2018 were retrospectively procured. Cases were enrolled in this study according to the criteria as follows: at least 18 years old; pathologically confirmed gastric adenocarcinoma; without autoimmune disease or other cancer types; without previous chemotherapy/radiotherapy. The pathologic diagnoses and characteristics were independently determined by at least two experienced pathologists. In addition, the clinicopathological and follow-up data were also collected.

**Independent cohort validation**

We used WES data generated from an independent cohort (n = 23) in order to determine whether our concise genomic classification model could be independently validated and has the potential to be applied in future clinical practice. Firstly, genomic features for each sample were extracted and used as model input for genomic classification prediction. Then the predicted subtype of each sample was obtained and subsequent clinical relevance was analyzed.

**References**

1. Frank E, Hall M, Trigg L, Holmes G, Witten IH. Data mining in bioinformatics using Weka. Bioinformatics. 2004;20(15):2479-2481.

2. Frank E, Hall M, Holmes G, Kirkby R, Pfahringer B, Witten IH, et al. Weka-A Machine Learning Workbench for Data Mining. Springer US. 2009.
